# Echinochrome A Reduces Colitis in Mice and Induces In Vitro Generation of Regulatory Immune Cells

**DOI:** 10.3390/md17110622

**Published:** 2019-10-31

**Authors:** Su-Jeong Oh, Yoojin Seo, Ji-Su Ahn, Ye Young Shin, Ji Won Yang, Hyoung Kyu Kim, Jin Han, Natalia P. Mishchenko, Sergey A. Fedoreyev, Valentin A. Stonik, Hyung-Sik Kim

**Affiliations:** 1 Department of Life Science in Dentistry, School of Dentistry, Pusan National University, Yangsan 50612, Korea; dhtnwjd26@naver.com (S.-J.O.); anjs08@naver.com (J.-S.A.); bubu3935@naver.com (Y.Y.S.); 2 Dental and Life Science Institute, Pusan National University, Yangsan 50612, Korea; amaicat24@naver.com (Y.S.); midnightnyou@naver.com (J.W.Y.); 3 National Research Laboratory for Mitochondrial Signaling, Department of Physiology, College of Medicine, Cardiovascular and Metabolic Disease Center (CMDC), Inje University, Busan 614-735, Korea; estrus74@gmail.com (H.K.K.); phyhanj@inje.ac.kr (J.H.); 4 G.B. Elyakov Pacific Institute of Bioorganic Chemistry, Far-Eastern Branch of the Russian Academy of Science, Vladivostok 690022, Russia; mischenkonp@mail.ru (N.P.M.); fedoreev-s@mail.ru (S.A.F.); stonik@piboc.dvo.ru (V.A.S.)

**Keywords:** echinochrome A, marine drugs, inflammatory bowel disease, regulatory T cells, macrophages

## Abstract

Echinochrome A (Ech A), a natural pigment extracted from sea urchins, is the active ingredient of a marine-derived pharmaceutical called ‘histochrome’. Since it exhibits several biological activities including anti-oxidative and anti-inflammatory effects, it has been applied to the management of cardiac injury and ocular degenerative disorders in Russia and its protective role has been studied for other pathologic conditions. In the present study, we sought to investigate the therapeutic potential of Ech A for inflammatory bowel disease (IBD) using a murine model of experimental colitis. We found that intravenous injection of Ech A significantly prevented body weight loss and subsequent lethality in colitis-induced mice. Interestingly, T cell proliferation was significantly inhibited upon Ech A treatment in vitro. During the helper T (Th) cell differentiation process, Ech A stimulated the generation regulatory T (Treg) cells that modulate the inflammatory response and immune homeostasis. Moreover, Ech A treatment suppressed the in vitro activation of pro-inflammatory M1 type macrophages, while inducing the production of M2 type macrophages that promote the resolution of inflammation and initiate tissue repair. Based on these results, we suggest that Ech A could provide a beneficial impact on IBD by correcting the imbalance in the intestinal immune system.

## 1. Introduction

Echinochrome A (Ech A) is a dark red pigment separated from sea urchin shell and spine and has a chemical structure of 6-ethyl-2,3,5,7,8-pentahydroxy-1,4-naphthoquinone [1,2]. As a main active component of a commercial therapeutic agent called ‘histochrome’, Ech A has been used for the treatment of cardiovascular disorders and ophthalmopathic complications in Russia [3,4,5]. Among the several biological benefits of Ech A, anti-oxidant and anti-inflammatory capacity is proposed as a major underlying therapeutic mechanism. Indeed, Ech A has been shown to attenuate the oxidative stress caused by reactive oxygen species (ROS) and cardiac toxic drugs, providing mitochondrial protection of cardiomyocyte [6]. Park et al. have reported similar observations showing that Ech A reduced both cellular and mitochondrial ROS levels of patient-derived cardiac progenitors during the oxidative stress situation [7]. The anti-oxidative and anti-viral activity of Ech A has also been proved in vitro using a tick-borne encephalitis virus and herpes simplex virus type 1-infected cell models [8]. The therapeutic potential of Ech A was also evaluated in an experimental gastric ulcer model where Ech A provided anti-ulcerogenic effects by increasing endogenous enzymatic and non-enzymatic antioxidant levels in vivo [9]. In another study, Ech A treatment could reduce ROS production and pro-inflammatory tumor necrosis factor-α (TNF-α) secretion in a rat model of acute uveitis induced by lipopolysaccharide injection [10]. These previous findings imply that Ech A could exert a wide range of therapeutic impacts on other oxidative stress-related and inflammatory pathologic conditions; however, the cell-type specific regulation of Ech A on the immune system, which consists of various innate and adaptive immune cells, has not been elucidated yet. 

Inflammatory bowel disease (IBD) is an intractable, chronic inflammatory disease of the digestive tract and Crohn’s disease (CD) and ulcerative colitis (UC) are the major types of IBD [11,12]. The etiology and pathogenic mechanisms of IBD remain largely unknown and both environmental factors and genetic factors combined with immunological dysfunction seem to drive IBD development. To attenuate the excessive immune response, advanced immunotherapy using immune-modulators such as inflammatory cytokine blockers has been used recently; however, the presence of non-responder and uncontrolled side effects are the common challenging issues when using immunotherapy [13,14]. Therefore, there has been an unmet need to develop novel therapeutics for the effective management of IBD. 

In this study, we investigated whether Ech A could exhibit a protective role in IBD progression using a chemical colitogen dextran sodium sulfate (DSS)-induced colitis mice model. To explore the therapeutic mechanism of Ech A, we also performed in vitro proliferation and polarization experiments with two major innate and adaptive immune cells, macrophage and CD4^+^ helper T cells (Th cells), respectively. Our in vivo findings suggest that Ech A could attenuate the clinical signs, as well as histological improvement, for the first time in a colitis model which represents IBD. More importantly, in vitro results demonstrate that the anti-inflammatory function of Ech A is manifested by, in part, inducing immunomodulatory effector cells, such as M2 macrophages and Treg cells.

## 2. Results and Discussion 

### 2.1. Ech A Treatment Exerted a Protective Effect against DSS-Induced Colitis In Vivo 

**Figure 1:**
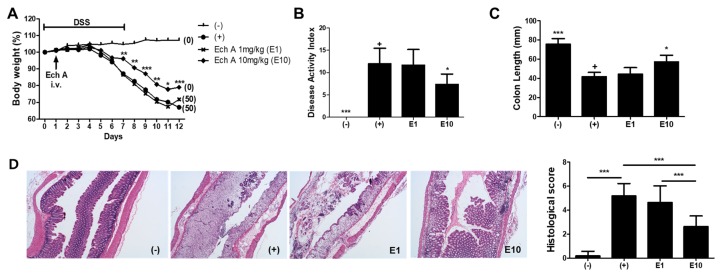
Echinochrome A (Ech A) administration provided therapeutic effects on DSS-induced colitis mice in a dose-dependent manner. (**A**) Body weight and survival rate monitoring results are shown. The body weight at day 0 considered was as 100%. Numbers in parentheses represent the percentage of dead mice. (**B**) Disease activity index score for colitis severity at day 12 was significantly reduced in the E10 group. (**C**) Colon length measurement results showing the protective role of Ech A against DSS-induced colonic damage. (**D**) The histopathological score of the colitis-affected and normal colon was evaluated with H&E stained tissue section. In total, six animals per group were used. Results are shown as mean ± SD. In (**B**) and (**C**), p-value significance was calculated by comparing other groups against the (+) group (marked as +). * *P* < 0.05, ** *P* < 0.01, *** *P* < 0.001.

To evaluate the therapeutic effects of Ech A on IBD, we gave a single intravenous (i.v) injection of Ech A or vehicle to DSS-induced colitis mice at day 1 and monitored the survival rate, body weight and disease activity index for 12 days. We found that a high dose (10 mg/kg; E10) of Ech A could significantly reduce body weight loss and increase the survival rate of colitis affected mice when compared with vehicle (+) and a low dose of Ech A (1 mg/kg; E1) treated groups (Figure 1A). According to the disease activity index score, clinical symptoms were also improved by Ech A treatment in a dose-dependent manner (Figure 1B). In the gross examination of the large intestine, pathologic shortening of the colon length due to colitis was reversed by the administration of Ech A (Figure 1C). H&E staining-based histopathological analysis revealed that typical pathologic signs of the colitis-affected damaged colon such as loss of epithelial structure and irregular morphology of crypts were ameliorated upon the administration of 10 mg/kg Ech A (Figure 1D). In addition, we found that excessive accumulation of immune cells within the epithelial and mesenchymal layer of the damaged colon was prevented upon Ech A treatment, suggesting that Ech A could exhibit anti-inflammatory roles in the in vivo mouse model (Figure 1D). Therefore, we concluded that Ech A could alleviate disease symptoms and play protective roles in DSS-induced colitis mice.

### 2.2. Ech A Suppressed the Proliferation of Human MNCs and T Lymphocytes In Vitro

**Figure 2:**
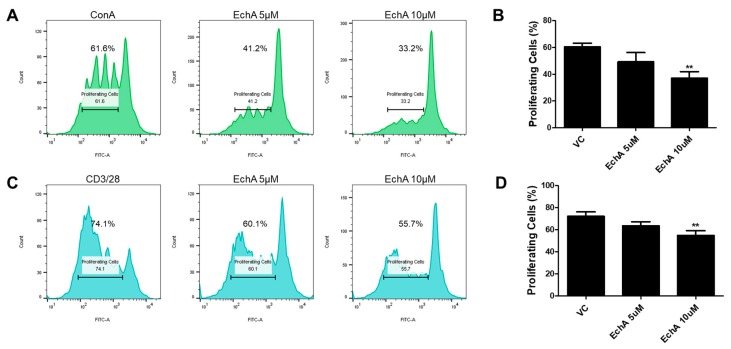
Ech A impact on mixed lymphocyte reaction (MLR) in vitro. CFSE-labeled human mononuclear cells (MNCs) were cultured for five days in the presence of mitogen or antibodies for CD3/28 with or without Ech A treatment, then the percentage of proliferated cells was evaluated using flow cytometry. The proliferation of both MNCs (**A**,**B**) and T cells (**C**,**D**) was reduced by Ech A treatment in a dose-dependent manner. VC, vehicle-treated control. ** *P* < 0.01. Results are shown as mean ± SD.

Based on the fact that excessive activation of the intestinal mucosal immune system triggered by increased epithelial permeability leads to aggravation of IBD progression [15,16], we next investigated whether Ech A could regulate the proliferation, differentiation and activation of various immune cells in vitro to explore the underlying therapeutic mechanisms of Ech A on the colitis model. First, we performed a mixed lymphocyte reaction (MLR) to evaluate the effect of Ech A on the expansion of human mononuclear cells (MNCs) and T lymphocytes. To stimulate in vitro proliferation, MNCs were treated with a non-specific mitogen concanavalin A (Con A) and cell proliferation capacity was evaluated using flow cytometry at day 5. Interestingly, the proliferation of Con A-treated MNCs was inhibited upon Ech A administration in a dose-dependent manner (Figure 2A,B). The percentage of proliferating cells in the control group was 60.4%, while it decreased to 49.3% and 37% upon 5 µM and 10 µM of Ech A treatment, respectively. Similarly, Ech A reduced the proliferation rate of CD3/CD28-stimulated T cells when compared with vehicle-treated samples (VC group: 72.2%; 5 µM Ech A group: 63.6%; 10 µM Ech A group: 54.9%) (Figure 2C,D), implying that Ech A could regulate immune cell proliferation in vitro.

### 2.3. Ech A Induced the Generation of Regulatory T Cells In Vitro

**Figure 3:**
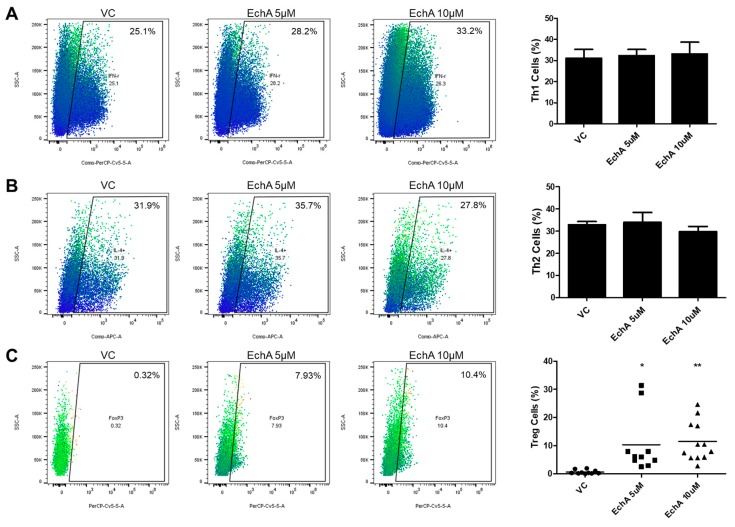
Ech A impact on Th cell polarization in vitro. (**A**,**B**) the percentages of IFN-γ ^+^ and IL-4^+^ cells among CD4^+^ Th cells were evaluated using flow cytometry to determine the induction ratio of Th1 and Th2 cells, respectively. No significant change was observed upon Ech A treatment. (**C**) Spontaneous generation of Foxp3^+^ Treg cells among CD4^+^ Th cells upon vehicle and Ech A treatment was assessed using flow cytometry. Ech A treatment led to an increase in Treg population compared to the vehicle-treated group. VC, vehicle-treated control. * *P* < 0.05, ** *P* < 0.001. Results are shown as mean ± SD.

It is well known that naive Th (Th0) cells can differentiate into various subtypes of active Th cells such as Th1, Th2 and Treg cells upon specific cues and each type of mature Th cell plays a distinct and pivotal role in various disease developments including IBD [17,18]. Therefore, next we isolated CD4^+^ naive Th0 cells from human cord blood then performed a differentiation experiment following the typical procedure with or without Ech A to evaluate the impact of Ech A on Th cell polarization. As shown in Figure 3, flow cytometry analysis on cell surface markers revealed that Ech A provided no significant impact on Th1/2 polarization in vitro (Figure 3A,B). On the other hand, Ech A treatment could stimulate the generation of Treg cells; indeed, approximately 10% of total Ech A-treated cells expressed the marker of the Treg cell marker Foxp3, while the spontaneous induction ratio of Treg cells were less than 1% on average (Figure 3C). 

### 2.4. Ech A Could Modulate the Polarization of Resting Macrophages into M1 and M2 Type In Vitro

**Figure 4:**
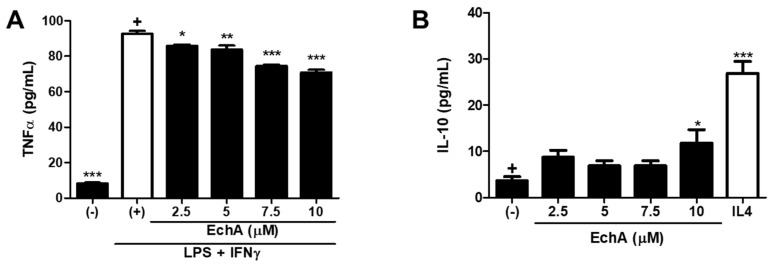
Ech A impact on M1/M2 macrophage polarization in vitro. (**A**) After five days of M1 induction, TNF-α concentration in the culture supernatant was measured by ELISA to estimate the M1 polarization efficiency. It is noted that Ech A prevented M1 polarization in vitro in a dose-dependent manner. (**B**) The IL-10 secretion level was determined using ELISA to evaluate the spontaneous M2 induction efficiency. Ech A treated macrophages were differentiated into M2 macrophages more effectively compared to vehicle-treated cells; (–), no induction control; (+), polarization induced control. The p-value significance was calculated by comparing other groups against the (+) and (–) groups (marked as +) in A and B, respectively. * *P* < 0.05, ** *P* < 0.01, *** *P* < 0.001. Results are shown as mean ± SD.

As a key component of the innate immune system, macrophages play important roles in the host defense reaction by mediating acute inflammatory response against danger signals and promoting stimulation of the adaptive immune system [19]. Typically, two main subtypes of macrophages have been described after polarization: Classically activated M1 type (M1) and alternative M2 type (M2) [20]. In general, M1 macrophages tend to mediate the excessive and persistent pro-inflammatory responses, while M2 macrophages are known to contribute to tissue regeneration and resolution of inflammation. Given that chronic inflammation with the M1/M2 polarization balance skewed toward the M1 phenotype is the major characteristic of IBD progression [21,22], modulation of macrophage activity could be another therapeutic target. Thus, we conducted an in vitro experiment to evaluate the influence of Ech A on M1/M2 differentiation. For M1 type polarization, phorbol 12-myristate-13-acetate (PMA)-treated THP1 macrophages were further stimulated with LPS and IFN-γ. As expected, M1 macrophages produced a high level of tumor necrosis factor-alpha (TNF-α) upon stimulation (Figure 4A). It was noted that Ech A could reduce M1-derived TNF-α secretion in a dose-dependent manner (Figure 4A). On the contrary, the basal secretion level of interleukin 10 (IL-10) was increased in the presence of Ech A, indicating that Ech A could induce the spontaneous M2 polarization in vitro (Figure 4B). 

From the in vivo observation, Ech A provided significant protection against experimental colitis as it alleviated disease severity and increased the survival rate of mice. In the histomorphological analysis of the colon, marked loss of villus-crypt structure accompanied with edematous erosion and granulation tissue formation was attenuated by Ech A injection. Ech A also decreased the degree of inflammatory cell infiltrates in a dose-dependent manner, suggesting that Ech A could exhibit an anti-inflammatory capacity in the IBD status.

Importantly, following in vitro Th cell differentiation, the experiment revealed that Ech A treatment could promote Treg generation without affecting the Th1 or Th2 populations. Considering that Treg cells function as a key immunomodulator that regulates activation of other Th cells, Ech A could contribute to correct the immunological imbalance in vivo by inducing the Treg population, although it does not directly regulate Th1 or Th2 polarization. Indeed, Tsai et al. have reported that a member of the lectin family galectin-3 prevents colitis progression while galectin-3 knockout mice suffered from more severe symptoms than controls, and the therapeutic impact of galectin-3 was abrogated by CD25 neutralization, implying the importance of Treg cells in this context [23]. Here we also demonstrated that Ech A could regulate the direction of macrophagic polarization toward M2 differentiation. M1 suppression and/or M2 induction strategies have proven beneficial for IBD treatment. Abron et al. have reported that the soy bean-derived natural agent genistein reduced the severity of DSS-induced colitis by transforming M1 macrophages into M2 type followed by the reduction of pro-inflammatory cytokine levels [24]. In addition, adipose-tissue-derived mesenchymal stem cells could provide protection in colitis mice models via TNF-α-induced gene/protein 6-mediated M2 induction [25]. In particular, the TNF-α inhibitory effect of Ech A could be directly applicable for IBD therapeutics considering that TNF-α expressing immune cells are increased in the intestinal tract of IBD patients and TNF-α blockers, as well as anti-TNF-α agents, have been approved in clinic for IBD treatment [26]. 

To further highlight the benefits of Ech A application, the underlying mechanisms of Ech A mediated immune cell fate regulation should be explored for the fundamental understanding of the Ech A therapeutic function. According to a recent study, Ech A could stimulate ex vivo expansion of CD34^+^ hematopoietic stem/progenitor cells (HSPC) with enhanced colony-forming capacity via suppression of intracellular ROS generation [27]. Mechanistically, ex vivo treatment of Ech A or anti-oxidant agent N-acetyl cysteine could not only inhibit oxidative stress-associated P38 MAPK activation but also increase Lyn/Src phosphorylation followed by activation of the PI3K-Akt signaling pathway, leading to high-quality HSPC expansion. Therefore, it would be worthwhile to investigate the therapeutic contribution of the anti-oxidative capacity of Ech A in IBD prevention in terms of HSPC-derived immune cell proliferation and differentiation. In addition, Ech A could bind directly to protein kinase C-iota (PKC i) and enhanced myocardial cell differentiation of mouse embryonic stem cells by antagonizing its activity [28]. Considering that the PKC family plays an important role in Th cell homeostasis [29,30], Ech A might regulate PKC activity to induce Treg population followed by M2 macrophagic activation. 

In conclusion, we demonstrate the therapeutic potential of Ech A for IBD for the first time. We also emphasize that Ech A could induce immunomodulatory effector cell generation such as Treg cells and M2 macrophages along with suppression of pro-inflammatory M1 macrophage, leading to attenuation of excessive inflammation. These data suggest that Ech A could exert immune-cell type specific regulatory roles in vitro and possibly in vivo. Therefore, our findings not only suggest Ech A as a novel potent therapeutic agent for incurable IBD but also contribute to expanding the therapeutic utility of Ech A for various inflammatory diseases.

## 3. Materials and Methods 

### 3.1. DSS-Induced Experimental Colitis Mice Modeling and Monitoring 

All experiments were approved by and followed the regulations of the Institute of Laboratory Animals Resources (PNU-2018-2034, Pusan National University). Experimental colitis was induced in mice by the addition of 3% (wt/vol) DSS (MP Biochemicals, Solon, OH, USA) in drinking water for 7 days as previously described [31]. Four groups were designed as positive (DSS, n = 6) or negative (no DSS, n = 6) controls and a low-dose (1 mg/kg, n = 6) and a high dose (10 mg/kg, n = 6) of Ech A treated group. Ech A was administrated at day 1 via i.v injection, while a vehicle (0.1% DMSO in PBS) was given to negative control mice instead. Total 12 day-long daily monitoring for body weight and survival rate was conducted. On day 12, the disease activity index of surviving animals was scored by assessing stool consistency, rectal bleeding and coat roughness (grade from 0 to 4), general activity and bedding contamination with stool and blood (graded from 0 to 2). All subjects were then sacrificed for the evaluation of gross pathologic severity (measuring colon length) and further histological analysis using H&E staining.

### 3.2. Mixed Leukocyte Reaction 

For MLR, human MNCs were labeled using the CellTrace CFSE cell labeling kit (Invitrogen, Grand Island, NY, USA) following the manufacturer`s instructions. Cells were then seeded in a 6-well plate at a density of 5 × 10^4^ /well and cultured in RPMI 1640 (Gibco, Grand Island, NY, USA) with supplement of 10% FBS (Gibco) and 100 U/mL penicillin/streptomycin (Gibco) in the presence of Con A (5 µg/mL for MNC proliferation) (Sigma, St. Lois, MO, USA) or anti-CD3/anti-CD28 (5 µg/mL and 2 µg/mL, respectively, for T cell proliferation) (eBioscience, San Diego, CA, USA) for 5 days. To assess the Ech A impact on cell proliferation, Ech A or vehicle was also treated during the culture period. After 5 days, cells were harvested and prepared for flow cytometry analysis.

### 3.3. Isolation and Culture of Human CD4^+^ Naïve Th (Th0) Cells 

This study was approved by the Institutional Review Board of Pusan National University (H-1802-006-063). MNCs were isolated from human cord blood provided by Busan/Ulsan/Gyeongnam Cord Blood Bank using HetaSep (Stem Cell Technologies, British Columbia, Canada) and Lymphoprep (Stem Cell Technologies) as per the manufacturer’s instructions. Total naive Th0 cells were then purified by negative selection with magnetic beads provided in the Human Naive CD4^+^ T Cell Isolation Kit II (Miltenyi Biotec, Bergisch Gladbach, Germany). Purified cells were determined for their purity (more than 95% CD4^+^ cells) and then plated in the density of 1 × 10^6^ cells/well in a 12-well culture plate and cultured in expansion media consisting of the ImmunoCult-XF T Cell Expansion Medium (Stem Cell Technologies), ImmunoCult Human CD3/CD28/CD2 T Cell Activator (Stem Cell Technologies) and IL-2 (Peprotech, Rocky Hill, NJ, USA).

### 3.4. Th1, Th2 and Treg Cell Differentiation 

For Th1 polarization, purified CD4^+^ Th0 cells were cultured in expansion media supplemented with IL-12 (10 ng/mL) (Peprotech) and anti-IL-4 neutralizing antibodies (5 μg/mL) (BD Bioscience, San Jose, CA, USA) for 5 days. For Th2 induction, purified CD4^+^ Th0 cells were cultured in expansion media with IL-4 (20 ng/mL) (Peprotech) and anti-IFN-γ neutralizing antibodies (5 μg/mL) (BioxCell, West Lebanon, NH, USA) for 5 days. To evaluate the spontaneous induction ratio of Treg cells, purified CD4^+^ Th0 cells were cultured in expansion media for 5 days without any lineage-specific stimulants. To assess the Ech A impact on Th cell polarization, Ech A or vehicle was also treated during the culture period.

### 3.5. Flow Cytometry Analysis 

To stimulate the production of lineage-specific markers, differentiated Th cells were treated with 50 ng/mL of PMA (Sigma) and 1 μM of Ionomycin (Sigma) with transport inhibitor GolgiStop (BD Biosciences) for 4 h. Cells were washed with PBS 4 times then incubated with FITC conjugated anti-human CD4 (BD Bioscience) at 4 °C for 30 min in the dark. After surface marker staining, cells were fixed and permeabilized using fixation and permeabilization buffer (BD Bioscience) then further stained with PerCP-Cy5.5-conjugated anti-human IFN-γ (BD Bioscience), APC-conjugated anti-human IL-4 (BD Bioscience), and PerCP-Cy5.5-conjugated anti-human FoxP3 (BD Bioscience) at 4 °C for 30 min in the dark. Nonspecific isotype-matched antibodies served as controls. Samples were analyzed with the BD FACS Verse flow cytometer (BD Biosciences) and data analysis was performed using FlowJo software.

### 3.6. Macrophage Polarization

THP1 cells were maintained in RPMI 1640 (Gibco, Grand Island, NY, USA) with a supplement of 10% FBS (Gibco) and 100 U/mL penicillin/streptomycin (Gibco). To induce monocytic differentiation, THP1 cells were plated at a density of 4 × 10^5^ /well on 6-well culture plate and pre-treated with PMA (Sigma) for 48 h. After PMA treatment, cells were stabilized for another 24 h in the maintenance media. Macrophages were then polarized in M1 macrophages by incubation with 20 ng/mL of IFN-γ (Peprotech) and 10 μg/mL of LPS (Sigma) for 5 days. PMA-pretreated cells were cultured within the maintenance media for 5 days without any lineage-specific stimulants for the spontaneous polarization towards M2 macrophages. To assess the Ech A impact on macrophage polarization, Ech A or vehicle was also treated during the culture period. Five days later, the culture supernatant was collected and secreted cytokine levels were estimated using human TNF-α and the IL-10 Duoset ELISA kit (R&D system, Abingdon, UK).

### 3.7. Data Analysis 

At least three individual experiments were conducted for in vitro experiments. Data are presented as mean ± standard error of the mean (SEM). All of the statistical comparisons were performed using one-way ANOVA followed by a Bonferroni post hoc test for multigroup comparisons using GraphPad Prism software (GraphPad Software, San Diego, CA, USA); p-values under 0.05 was considered statistically significant.
